# *rac*-7-Oxabicyclo­[2.2.1]heptane-2,3-dicarboxylic acid–2-amino-1,3,4-thia­diazole–water (1/1/1)

**DOI:** 10.1107/S1600536809021825

**Published:** 2009-06-17

**Authors:** Na Wang, Qiu-Yue Lin, Yan-Jun Wang

**Affiliations:** a Zhejiang Key Laboratory for Reactive Chemistry on Solid Surfaces, Institute of Physical Chemistry, Zhejiang Normal University, Jinhua, Zhejiang 321004, People’s Republic of China, and, College of Chemistry and Life Science, Zhejiang Normal University, Jinhua 321004, Zhejiang, People’s Republic of China

## Abstract

The title compound, C_8_H_10_O_5_·C_2_H_3_N_3_S·H_2_O, was synthesized by the reaction of 2-amino-1,3,4-thia­diazole with norcantharidin. The crystal structure is stabilized by N—H⋯O, N—H⋯N, O—H⋯O and O—H⋯N hydrogen bonds. In addition, weak π–π inter­actions are observed between symmetry-related thia­diazole ring systems [centroid–centroid distance = 3.9110 (3) Å, inter­planar spacing = 3.4845 Å].

## Related literature

7-Oxabicyclo­[2.2.1]heptane-2,3-dicarboxylic anhydride (nor­cantharidin) is a lower toxicity anti­cancer drug, see: Shimi & Zaki (1982 ▶).
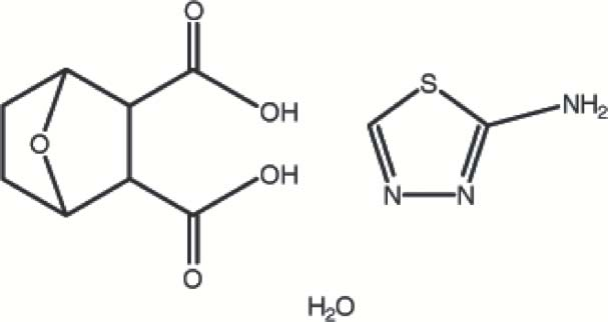


## Experimental

### 

#### Crystal data

C_8_H_10_O_5_·C_2_H_3_N_3_S·H_2_O
*M* *_r_* = 305.31
Monoclinic, 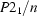

*a* = 5.7678 (5) Å
*b* = 18.4267 (15) Å
*c* = 12.7546 (11) Å
β = 101.336 (6)°
*V* = 1329.1 (2) Å^3^
*Z* = 4
Mo *K*α radiation
μ = 0.27 mm^−1^
*T* = 296 K
0.30 × 0.16 × 0.09 mm

#### Data collection

Bruker APEXII area-detector diffractometer
Absorption correction: multi-scan (*SADABS*; Sheldrick, 1996 ▶) *T*_min_ = 0.949, *T*_max_ = 0.977
10820 measured reflections
2995 independent reflections
2026 reflections with *I* > 2σ(*I*)
*R*_int_ = 0.040

#### Refinement

*R*[*F* ^2^ > 2σ(*F* ^2^)] = 0.046
*wR*(*F* ^2^) = 0.133
*S* = 1.05
2995 reflections
187 parameters
3 restraints
H atoms treated by a mixture of independent and constrained refinement
Δρ_max_ = 0.30 e Å^−3^
Δρ_min_ = −0.29 e Å^−3^

### 

Data collection: *APEX2* (Bruker, 2006 ▶); cell refinement: *SAINT* (Bruker, 2006 ▶); data reduction: *SAINT*; program(s) used to solve structure: *SHELXS97* (Sheldrick, 2008 ▶); program(s) used to refine structure: *SHELXL97* (Sheldrick, 2008 ▶); molecular graphics: *SHELXTL* (Sheldrick, 2008 ▶); software used to prepare material for publication: *SHELXL97*.

## Supplementary Material

Crystal structure: contains datablocks I, global. DOI: 10.1107/S1600536809021825/at2800sup1.cif

Structure factors: contains datablocks I. DOI: 10.1107/S1600536809021825/at2800Isup2.hkl

Additional supplementary materials:  crystallographic information; 3D view; checkCIF report

## Figures and Tables

**Table 1:** Hydrogen-bond geometry (Å, °)

*D*—H⋯*A*	*D*—H	H⋯*A*	*D*⋯*A*	*D*—H⋯*A*
N1—H1*A*⋯O4^i^	0.86	2.11	2.930 (3)	160
N1—H1*C*⋯N3^ii^	0.86	2.15	2.994 (3)	166
N1—H1*C*⋯N2^ii^	0.86	2.69	3.519 (3)	161
O2—H2*A*⋯O1*W*^iii^	0.82	1.81	2.626 (2)	176
O5—H5*B*⋯N2^iv^	0.82	1.85	2.664 (2)	172
O1*W*—H1*WA*⋯O3^v^	0.859 (17)	1.910 (17)	2.766 (2)	175 (3)
O1*W*—H1*WB*⋯O4^vi^	0.819 (17)	2.51 (3)	3.151 (3)	137 (3)
O1*W*—H1*WB*⋯O1^vi^	0.819 (17)	2.55 (3)	3.061 (3)	122 (3)

Symmetry codes: (i) 
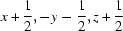
; (ii) 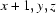; (iii) 
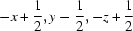
; (iv) 
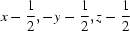
; (v) 
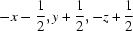
; (vi) 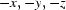.

**Fig. 1.**
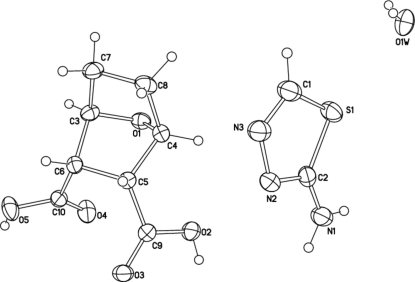
A view of the molecule of (I) showing the atom-labelling scheme with displacement ellipsoids drawn at the 30% probability.

## supplementary crystallographic information

### Comment

7-Oxabicyclo[2.2.1]heptane-2,3-dicarboxylic anhydride (norcantharidin) derived from cantharidin is a lower toxicity anticancer drug (Shimi & Zaki, 1982). The title compound was synthesized by the reaction of 2-amino-1,3,4-thiadiazole with 7-oxabicyclo[2.2.1]heptane-2,3-dicarboxylic anhydride (norcantharidin). In this paper, we reports its structure.

X-ray crystallography measurement confirmed the molecular structure and the atom connectivity for the title compound (Fig. 1). The crystal structure is stabilized by N—H···O, N—H···N, O—H···O and O—H···N hydrogen bonds (Table 1). Further, weak π–π interactions are observed between symmetry related thiadiazole ring systems [centroid-centroid distance of 3.9110 (3)Å and interplanar spacing of 3.4845 Å].

### Experimental

7-Oxabicyclo[2.2.1]heptane-2,3-dicarboxylic anhydride and 2-amino-1,3,4-thiadiazole were dissolved in tetrahydrofuran and the mixture was stirred for 6 h at room temperature. The clear solution was left undisturbed for days to give colourless crystals of the compound.

### Refinement

The H atoms bonded to C and N atoms were positioned geometrically and refined using ariding model [C—H =0.93- 0.98 Å, N—H = 0.86 Å and O—H = 0.82 Å and *U*_iso_(H) = 1.2 or 1.5*U*_eq_(C,N,O)]. The H atoms of the water molecule were located in a difference Fourier maps and refined with O—H distance restraints of 0.85 (2) and *U*_iso_(H) = 1.5*U*_eq_(O).

### Crystal data

**Table:**

C_8_H_10_O_5_·C_2_H_3_N_3_S·H_2_O	*F*(000) = 640
*M**_r_* = 305.31	*D*_x_ = 1.526 Mg m^−^^3^
Monoclinic, *P*2_1_/*n*	Mo *K*α radiation, λ = 0.71073 Å
Hall symbol: -P 2yn	Cell parameters from 1905 reflections
*a* = 5.7678 (5) Å	θ = 2.0–27.6°
*b* = 18.4267 (15) Å	µ = 0.27 mm^−^^1^
*c* = 12.7546 (11) Å	*T* = 296 K
β = 101.336 (6)°	Block, colourless
*V* = 1329.1 (2) Å^3^	0.30 × 0.16 × 0.09 mm
*Z* = 4	

### Data collection

**Table:**

Bruker APEXII area-detector diffractometer	2995 independent reflections
Radiation source: fine-focus sealed tube	2026 reflections with *I* > 2σ(*I*)
graphite	*R*_int_ = 0.040
ω scans	θ_max_ = 27.6°, θ_min_ = 2.0°
Absorption correction: multi-scan (*SADABS*; Sheldrick, 1996)	*h* = −6→7
*T*_min_ = 0.949, *T*_max_ = 0.977	*k* = −24→23
10820 measured reflections	*l* = −16→15

### Refinement

**Table:**

Refinement on *F*^2^	Primary atom site location: structure-invariant direct methods
Least-squares matrix: full	Secondary atom site location: difference Fourier map
*R*[*F*^2^ > 2σ(*F*^2^)] = 0.046	Hydrogen site location: inferred from neighbouring sites
*wR*(*F*^2^) = 0.133	H atoms treated by a mixture of independent and constrained refinement
*S* = 1.05	*w* = 1/[σ^2^(*F*_o_^2^) + (0.062*P*)^2^ + 0.3174*P*] where *P* = (*F*_o_^2^ + 2*F*_c_^2^)/3
2995 reflections	(Δ/σ)_max_ = 0.001
187 parameters	Δρ_max_ = 0.30 e Å^−^^3^
3 restraints	Δρ_min_ = −0.29 e Å^−^^3^

### Special details

**Table:**

Geometry. All e.s.d.'s (except the e.s.d. in the dihedral angle between two l.s. planes) are estimated using the full covariance matrix. The cell e.s.d.'s are taken into account individually in the estimation of e.s.d.'s in distances, angles and torsion angles; correlations between e.s.d.'s in cell parameters are only used when they are defined by crystal symmetry. An approximate (isotropic) treatment of cell e.s.d.'s is used for estimating e.s.d.'s involving l.s. planes.
Refinement. Refinement of *F*^2^ against ALL reflections. The weighted *R*-factor *wR* and goodness of fit *S* are based on *F*^2^, conventional *R*-factors *R* are based on *F*, with *F* set to zero for negative *F*^2^. The threshold expression of *F*^2^ > σ(*F*^2^) is used only for calculating *R*-factors(gt) *etc*. and is not relevant to the choice of reflections for refinement. *R*-factors based on *F*^2^ are statistically about twice as large as those based on *F*, and *R*- factors based on ALL data will be even larger.

### Fractional atomic coordinates and isotropic or equivalent isotropic displacement parameters (Å^2^)

**Table:**

	*x*	*y*	*z*	*U*_iso_*/*U*_eq_	
C1	−0.2976 (5)	0.05357 (14)	0.3265 (2)	0.0537 (7)	
H1B	−0.3880	0.0947	0.3053	0.064*	
C2	−0.0094 (4)	−0.03835 (13)	0.37115 (19)	0.0401 (6)	
C3	−0.7049 (4)	−0.14263 (12)	−0.02775 (19)	0.0371 (5)	
H3A	−0.7478	−0.1570	−0.1031	0.044*	
C4	−0.4729 (4)	−0.10527 (12)	0.11367 (19)	0.0378 (5)	
H4A	−0.3228	−0.0881	0.1563	0.045*	
C5	−0.5597 (4)	−0.17663 (11)	0.15371 (18)	0.0308 (5)	
H5A	−0.6555	−0.1649	0.2069	0.037*	
C6	−0.7289 (4)	−0.20482 (11)	0.05131 (18)	0.0320 (5)	
H6A	−0.8916	−0.2080	0.0630	0.038*	
C7	−0.8388 (4)	−0.07593 (13)	−0.0002 (2)	0.0436 (6)	
H7A	−0.9932	−0.0888	0.0135	0.052*	
H7B	−0.8573	−0.0399	−0.0566	0.052*	
C8	−0.6732 (4)	−0.04919 (12)	0.1016 (2)	0.0451 (6)	
H8A	−0.7506	−0.0499	0.1626	0.054*	
H8B	−0.6158	−0.0006	0.0925	0.054*	
C9	−0.3734 (4)	−0.23059 (12)	0.20304 (18)	0.0339 (5)	
C10	−0.6556 (4)	−0.27519 (12)	0.00717 (19)	0.0357 (5)	
S1	−0.00230 (12)	0.04890 (3)	0.32282 (6)	0.0514 (2)	
N1	0.1766 (3)	−0.08184 (12)	0.39063 (18)	0.0534 (6)	
H1A	0.1631	−0.1250	0.4145	0.064*	
H1C	0.3110	−0.0670	0.3794	0.064*	
N2	−0.2197 (3)	−0.05840 (10)	0.38689 (17)	0.0431 (5)	
N3	−0.3854 (4)	−0.00394 (11)	0.36078 (19)	0.0511 (6)	
O1	−0.4621 (3)	−0.12182 (8)	0.00484 (13)	0.0399 (4)	
O1W	0.3491 (3)	0.19399 (12)	0.19534 (16)	0.0573 (5)	
O2	−0.1540 (3)	−0.20904 (9)	0.21051 (15)	0.0470 (5)	
H2A	−0.0641	−0.2405	0.2405	0.071*	
O3	−0.4251 (3)	−0.28854 (9)	0.23715 (14)	0.0453 (4)	
O4	−0.4568 (3)	−0.28772 (9)	−0.00388 (16)	0.0527 (5)	
O5	−0.8343 (3)	−0.31955 (9)	−0.02355 (17)	0.0575 (5)	
H5B	−0.7870	−0.3568	−0.0474	0.086*	
H1WA	0.213 (4)	0.1986 (18)	0.212 (2)	0.086*	
H1WB	0.317 (5)	0.2024 (19)	0.1311 (15)	0.086*	

### Atomic displacement parameters (Å^2^)

**Table:**

	*U*^11^	*U*^22^	*U*^33^	*U*^12^	*U*^13^	*U*^23^
C1	0.0577 (16)	0.0398 (14)	0.064 (2)	0.0017 (11)	0.0119 (14)	0.0069 (13)
C2	0.0431 (13)	0.0412 (13)	0.0358 (14)	−0.0098 (10)	0.0075 (10)	−0.0022 (10)
C3	0.0419 (13)	0.0369 (12)	0.0311 (13)	0.0077 (9)	0.0038 (10)	−0.0009 (10)
C4	0.0383 (12)	0.0342 (12)	0.0394 (14)	−0.0035 (9)	0.0041 (10)	0.0000 (10)
C5	0.0310 (11)	0.0334 (11)	0.0289 (12)	0.0019 (8)	0.0081 (9)	0.0004 (9)
C6	0.0280 (11)	0.0316 (11)	0.0371 (14)	0.0013 (8)	0.0081 (9)	−0.0036 (9)
C7	0.0443 (13)	0.0368 (12)	0.0486 (16)	0.0118 (10)	0.0062 (11)	0.0015 (11)
C8	0.0606 (16)	0.0306 (12)	0.0448 (16)	0.0032 (10)	0.0119 (12)	−0.0033 (11)
C9	0.0339 (12)	0.0390 (12)	0.0300 (13)	0.0015 (9)	0.0094 (9)	−0.0002 (10)
C10	0.0366 (13)	0.0334 (12)	0.0365 (14)	0.0016 (9)	0.0058 (10)	−0.0027 (9)
S1	0.0575 (4)	0.0399 (4)	0.0583 (5)	−0.0095 (3)	0.0150 (3)	0.0073 (3)
N1	0.0475 (12)	0.0419 (12)	0.0697 (17)	−0.0015 (9)	0.0089 (11)	0.0107 (11)
N2	0.0455 (11)	0.0354 (10)	0.0480 (13)	−0.0044 (8)	0.0085 (9)	0.0048 (9)
N3	0.0462 (12)	0.0434 (12)	0.0625 (15)	0.0023 (9)	0.0083 (11)	0.0072 (11)
O1	0.0417 (9)	0.0391 (9)	0.0425 (10)	0.0032 (7)	0.0170 (7)	0.0082 (7)
O1W	0.0381 (10)	0.0837 (14)	0.0524 (12)	−0.0079 (9)	0.0140 (9)	−0.0121 (11)
O2	0.0315 (9)	0.0499 (10)	0.0589 (12)	0.0004 (7)	0.0068 (8)	0.0103 (8)
O3	0.0374 (9)	0.0435 (9)	0.0558 (12)	0.0049 (7)	0.0108 (8)	0.0171 (8)
O4	0.0406 (10)	0.0447 (10)	0.0747 (14)	0.0032 (7)	0.0163 (9)	−0.0214 (9)
O5	0.0411 (10)	0.0362 (9)	0.0954 (16)	−0.0057 (7)	0.0137 (9)	−0.0213 (10)

### Geometric parameters (Å, °)

**Table:**

C1—N3	1.287 (3)	C6—H6A	0.9800
C1—S1	1.715 (3)	C7—C8	1.534 (3)
C1—H1B	0.9300	C7—H7A	0.9700
C2—N2	1.320 (3)	C7—H7B	0.9700
C2—N1	1.323 (3)	C8—H8A	0.9700
C2—S1	1.725 (2)	C8—H8B	0.9700
C3—O1	1.433 (3)	C9—O3	1.212 (3)
C3—C7	1.529 (3)	C9—O2	1.311 (3)
C3—C6	1.550 (3)	C10—O4	1.205 (3)
C3—H3A	0.9800	C10—O5	1.313 (3)
C4—O1	1.434 (3)	N1—H1A	0.8600
C4—C5	1.530 (3)	N1—H1C	0.8600
C4—C8	1.535 (3)	N2—N3	1.381 (3)
C4—H4A	0.9800	O1W—H1WA	0.859 (17)
C5—C9	1.508 (3)	O1W—H1WB	0.819 (17)
C5—C6	1.558 (3)	O2—H2A	0.8200
C5—H5A	0.9800	O5—H5B	0.8200
C6—C10	1.507 (3)		
			
N3—C1—S1	115.3 (2)	C5—C6—H6A	110.7
N3—C1—H1B	122.4	C3—C7—C8	101.18 (17)
S1—C1—H1B	122.4	C3—C7—H7A	111.5
N2—C2—N1	122.5 (2)	C8—C7—H7A	111.5
N2—C2—S1	113.71 (18)	C3—C7—H7B	111.5
N1—C2—S1	123.80 (18)	C8—C7—H7B	111.5
O1—C3—C7	103.14 (18)	H7A—C7—H7B	109.4
O1—C3—C6	102.49 (17)	C7—C8—C4	101.53 (18)
C7—C3—C6	109.34 (19)	C7—C8—H8A	111.5
O1—C3—H3A	113.6	C4—C8—H8A	111.5
C7—C3—H3A	113.6	C7—C8—H8B	111.5
C6—C3—H3A	113.6	C4—C8—H8B	111.5
O1—C4—C5	102.67 (17)	H8A—C8—H8B	109.3
O1—C4—C8	102.77 (18)	O3—C9—O2	122.9 (2)
C5—C4—C8	108.84 (18)	O3—C9—C5	121.68 (19)
O1—C4—H4A	113.8	O2—C9—C5	115.39 (19)
C5—C4—H4A	113.8	O4—C10—O5	123.7 (2)
C8—C4—H4A	113.8	O4—C10—C6	123.5 (2)
C9—C5—C4	116.94 (17)	O5—C10—C6	112.67 (18)
C9—C5—C6	114.05 (17)	C1—S1—C2	86.76 (12)
C4—C5—C6	101.48 (17)	C2—N1—H1A	120.0
C9—C5—H5A	108.0	C2—N1—H1C	120.0
C4—C5—H5A	108.0	H1A—N1—H1C	120.0
C6—C5—H5A	108.0	C2—N2—N3	111.89 (19)
C10—C6—C3	108.99 (18)	C1—N3—N2	112.4 (2)
C10—C6—C5	115.12 (17)	C3—O1—C4	96.42 (15)
C3—C6—C5	100.22 (17)	H1WA—O1W—H1WB	101 (2)
C10—C6—H6A	110.7	C9—O2—H2A	109.5
C3—C6—H6A	110.7	C10—O5—H5B	109.5
			
O1—C4—C5—C9	90.1 (2)	C6—C5—C9—O3	−62.0 (3)
C8—C4—C5—C9	−161.49 (19)	C4—C5—C9—O2	2.8 (3)
O1—C4—C5—C6	−34.65 (19)	C6—C5—C9—O2	120.9 (2)
C8—C4—C5—C6	73.8 (2)	C3—C6—C10—O4	65.8 (3)
O1—C3—C6—C10	−86.2 (2)	C5—C6—C10—O4	−45.8 (3)
C7—C3—C6—C10	164.91 (18)	C3—C6—C10—O5	−110.5 (2)
O1—C3—C6—C5	35.06 (19)	C5—C6—C10—O5	137.9 (2)
C7—C3—C6—C5	−73.9 (2)	N3—C1—S1—C2	0.4 (2)
C9—C5—C6—C10	−10.1 (3)	N2—C2—S1—C1	−0.1 (2)
C4—C5—C6—C10	116.51 (19)	N1—C2—S1—C1	179.7 (2)
C9—C5—C6—C3	−126.86 (18)	N1—C2—N2—N3	−179.9 (2)
C4—C5—C6—C3	−0.22 (19)	S1—C2—N2—N3	−0.1 (3)
O1—C3—C7—C8	−34.6 (2)	S1—C1—N3—N2	−0.5 (3)
C6—C3—C7—C8	73.9 (2)	C2—N2—N3—C1	0.4 (3)
C3—C7—C8—C4	0.3 (2)	C7—C3—O1—C4	56.17 (19)
O1—C4—C8—C7	33.9 (2)	C6—C3—O1—C4	−57.40 (18)
C5—C4—C8—C7	−74.5 (2)	C5—C4—O1—C3	57.35 (18)
C4—C5—C9—O3	179.9 (2)	C8—C4—O1—C3	−55.63 (18)

### Hydrogen-bond geometry (Å, °)

**Table:**

*D*—H···*A*	*D*—H	H···*A*	*D*···*A*	*D*—H···*A*
N1—H1A···O4^i^	0.86	2.11	2.930 (3)	160
N1—H1C···N3^ii^	0.86	2.15	2.994 (3)	166
N1—H1C···N2^ii^	0.86	2.69	3.519 (3)	161
O2—H2A···O1W^iii^	0.82	1.81	2.626 (2)	176
O5—H5B···N2^iv^	0.82	1.85	2.664 (2)	172
O1W—H1WA···O3^v^	0.86 (2)	1.91 (2)	2.766 (2)	175 (3)
O1W—H1WB···O4^vi^	0.82 (2)	2.51 (3)	3.151 (3)	137 (3)
O1W—H1WB···O1^vi^	0.82 (2)	2.55 (3)	3.061 (3)	122 (3)

Symmetry codes: (i) *x*+1/2, −*y*−1/2, *z*+1/2; (ii) *x*+1, *y*, *z*; (iii) −*x*+1/2, *y*−1/2, −*z*+1/2; (iv) *x*−1/2, −*y*−1/2, *z*−1/2; (v) −*x*−1/2, *y*+1/2, −*z*+1/2; (vi) −*x*, −*y*, −*z*.
